# Similar Biochemical Signatures and Prion Protein Genotypes in Atypical Scrapie and Nor98 Cases, France and Norway

**DOI:** 10.3201/eid1301.060393

**Published:** 2007-01

**Authors:** Jean-Noël Arsac, Olivier Andreoletti, Jean-Marc Bilheude, Caroline Lacroux, Sylvie L. Benestad, Thierry Baron

**Affiliations:** * Agence Française de Sécurité Sanitaire des Aliments, Lyon, France; † Ecole Nationale Vétérinaire de Toulouse, Toulouse, France; ‡ Bio-Rad, Marnes-la-Coquette, France; § National Veterinary Institute, Oslo, Norway

**Keywords:** Scrapie, prion, genotype, sheep, goat, Western blot, research

## Abstract

Isolates of atypical scrapie recently identified in sheep and goats in France were compared with Nor98 isolates reported in Norway. Western blot methods for characterization of the protease-resistant prion protein showed that all these isolates shared a unique biochemical signature: 5 groups of bands, including a characteristic band of apparent low molecular weight (11 kDa). This pattern could originate from the presence of 3 different protease cleavage products, including the 11 kDa most likely cleaved at both N- and C-sides of the protein. Genetic data, which strongly suggested the higher susceptibility of AHQ and AF_141_RQ animals in French cases, resembled earlier data from Nor98 scrapie.

## Abstract 2

Similarities raise questions regarding the origin of these recently described cases.

Transmissible spongiform encephalopathies (TSE) are neurodegenerative disorders that occur in sheep and goats (scrapie), cattle (bovine spongiform encephalopathy [BSE]), or humans (Creutzfeldt-Jakob disease). The biochemical marker of the disease is currently considered to be the accumulation of an abnormal isoform (PrPres) of the normal cellular prion protein (PrPc). PrPres can be identified by its partial resistance to proteases and insolubility in detergents. Classically, after pK treatment and Western blot (WB), PrPres exhibits a typical 3-band pattern comprising 18–30 kDa, whereas PrPc is totally digested (*1*,*2*).

Ovine susceptibility to scrapie is largely controlled by polymorphisms at the PrP gene (*prnp)*. The major polymorphisms associated with susceptibility or resistance are located at codons 136 (A or V), 154 (R or H), and 171 (R, Q, or H) (*3*,*4*). V^136^R^154^Q^171^/VRQ, ARQ/VRQ, and ARQ/ARQ PrP animals are considered the most susceptible to scrapie, whereas homozygous or heterozygous AHQ and heterozygous ARR animals show only marginal susceptibility (*5*). ARR/ARR sheep are considered to be the more resistant (*4*,*6*), but after oral challenge with BSE agent they can accumulate PrPres in the spleen (*7*).

In 1998, a novel and unusual TSE type (called Nor98) was identified in sheep in Norway (*8*). A large proportion of animals were carriers of AHQ and AF_141_RQ alleles (*9*). The PrP WB signature in these cases differed from the known scrapie profile; the classic 3-band WB pattern was replaced by a multiband pattern with a prominent band of low molecular mass (≈12 kDa).

Since 2002, an active surveillance program for TSE has been implemented in small ruminants in European Union (EU) countries. As a result of this program, unusual TSE isolates were rapidly identified in sheep and goats in France, Germany (*10*,*11*), and Great Britain (*12*). These atypical TSE isolates had the following characteristics: 1) they came from sheep carrying PrP alleles reportedly associated with resistance to TSEs; 2) their rapid diagnostic test results based on PrPres detection showed discrepancies; and 3) they could not be readily confirmed by recommended Office International des Epizooties diagnostic methods (*10*). In this study we investigated a panel of 54 French atypical isolates from sheep (n = 51) and goats (n = 3) and compared their PrP genotypes and advanced PrPres biochemical signatures (WB electrophoretic mobility, epitope mapping, and PrPres deglycosylation pattern) with those of Nor98 cases.

## Materials and Methods

### Small Ruminant Isolates

The biologic samples (Table) consisted of 54 brain stems from 51 sheep from France and 3 goats previously classified as having atypical scrapie and collected during the active surveillance program between 2002 and 2004. These samples were compared with Nor98 samples from 4 Norwegian sheep.

**Table:** Atypical, Nor98, and classic scrapie isolates from sheep and goats and distribution of genotypes*

		Sheep isolates			Goat isolates	
Genotype		Atypical, n = 51 (no. of fully characterized isolates)	Nor98, n = 4	Classic, n = 74	Atypical, n = 3	Classic, n = 60
AF_141_RQ/	ARR	16 (3)	1 (0)	–	–	–
	ARQ	9 (3)	–	5	–	–
	AF_141_RQ	7 (3)	1 (0)	–	–	–
	VRQ	3 (0)	–	4	–	–
AHQ/	ARR	2 (1)	–	–	–	–
	AHQ	2 (0)	2 (1)	–	1 (1)	–
	ARQ	1 (0)	–	–	1 (1)	–
	ARH	1 (1)	–	–	–	–
	VRQ	1 (0)	–	–	–	–
ARR/	ARR	6 (3)	–	–	–	–
ARQ/	ARR	2 (2)	–	–	–	–
	ARQ	–	–	29	1 (0)	60
	ARH	–	–	13	–	–
ARH/	ARH	1 (1)	–	3	–	–
VRQ/	ARQ	–	–	10	–	–
	VRQ	–	–	9	–	–
	ARH	–	–	1	–	–

*Amino acids encoded at positions 136, 141, 154, and 171 are indicated.

The *prnp* polymorphisms from these atypical cases were compared with a panel from animals with classic scrapie (74 clinically affected sheep obtained from 60 flocks between 2000 and 2002 and 60 scrapie-positive goats obtained from 13 flocks between 2002 and 2004. The animals with classic scrapie were not matched for age, breed, or flock structure with animals with atypical scrapie.

### *prnp* ORF Sequencing

In each case, DNA was directly recovered from brain stem 30 mg) by using a commercial DNA extraction kit (Qiaprep DNeasy Minikit (QIAGEN, Courtaboeuf, France) according to manufacturer's recommendations. The complete open reading frame (ORF) sequence of the *prnp* gene was determined by sequencing both strands of 2 overlapping PCR fragments covering the complete ovine *prnp* ORF (primer 1F: GTGGGCATTTGATGCTGACAC, primer 1R: TGGTTGGGGTAACGGTACATG, Tm1 59°C, primer 2F: TCAGCCCCATGGTGGTGGCT, primer 2R: CTGCAGGTAGACACTCCCTCC, Tm2 61°C). The same primers were used for the goat samples.

The PCR products were amplified for 35 cycles (extension time 45 s) and allowed to migrate on 1% agarose gel. They were then purified, and both strands were sequenced. The appropriate software (SemanII, DNAstar, Monluçon, France) was then used to reconstitute the ORF sequence and align it with reference sequences from both ovine and caprine species.

### PrPres Purification and Western Blotting

Samples were examined by TeSeE WB (Bio-Rad, Marnes la Coquette, France), according to manufacturer’s recommendations. Briefly, 20% brain homogenate was incubated with pK and detergent solution for 10 min at 37°C before buffer B was added. Samples were then centrifuged at 15,000× *g* for 7 min and the pellet solubilized by incubation at 100°C for 5 min in 100 μL Laemmli solution completed (Bio-Rad) with 5% (v/v) β-mercaptoethanol and 2% (w/v) sodium dodecyl sulfate (SDS). Samples were centrifuged at 15,000× *g* for 15 min. The supernatants were then heated at 100°C for 5 min and subjected to electrophoresis. The undiluted sample (equivalent to 15 mg of tissue) was loaded onto homemade acrylamide SDS-polyacrylamide gel (PAGE).

PAGE gels (15% resolving gel and 4% stacking gel) migrated for 60 min at 200 V. The proteins were transferred onto a polyvinylidene difluoride membrane at 115 V for 60 min. The membrane was soaked successively with phosphate-buffered saline (PBS), ethanol, and distilled water; saturated with blocking solution for 30 min; and then incubated for 30 min at room temperature with Sha 31 (4 μg/mL in PBS-Tween [PBST]) against the YEDRYYRE (148–155) ovine PrP sequence (*13*). The membrane was then washed with PBST and incubated for 20 min with goat anti-mouse immunoglobulin G (IgG) antibody conjugated with horseradish peroxidase diluted 1:10 in PBST. It was the subjected to the enhanced chemiluminescence WB detection (Amersham or Supersignal, Pierce, Orsay, France) and visualized by using the Versa Doc image analysis system (Bio-Rad).

### MW Determination

A panel of 20 of these samples from 17 French sheep with atypical cases, 2 French goats with atypical cases, and a Norwegian sheep with Nor98 (Table) were selected for detailed molecular characterization. The apparent molecular weights (MWs) were determined with a protein standard (B2787; Sigma, Saint Louis, MO, USA). Each band was measured (apparent MWs and proportions) by using Quantity One software (Bio-Rad).

### Epitope Mapping

Different monoclonal antibodies (MAbs) were used for detection of PrPres fragments: Sha 31 (4 μg/mL in PBST), P4 (1/2,500 in PBST) (R-Biopharm, Saint-Didier Au Mont d’Or, France), 4F2 (1/2,500 in PBST), and 99/97.6.1 (1/2,500 in PBST). They recognized the following respective ovine sequences: YEDRYYRE (148–155) (*13*), WGQGGSH (93–99) (*14*),QPHGGGW (62–93), and 99/97.6.1 YQRE (221–224) (J. Langeveld, unpub. Pepscan data). The membranes were washed and then incubated with peroxidase-labeled conjugates against mouse Ig (1/2,500 in PBST) (Ozyme, Saint Quentin/Yvelines, France).

### Deglycosylation Experiments

Deglycosylation experiments were performed on 6 French and 1 Nor98 isolates with PNGaseF, following the Ozyme manufacturer’s instructions (P0704S) and TeSeE WB protocol for sample purification. Briefly, after PrPres purification, the pellet was solubilized by incubating at 100°C for 10 min in glycoprotein denaturing buffer 1× instead of Laemmli (Bio-Rad) solution. Samples were treated with PNGase F for 1 h at 37°C (reaction buffer 1×, 10% NP-40, PNGase F); buffer B was then added. Samples were centrifuged at 15,000× *g* for 7 min, and the pellets were solubilized in 100 μL completed Laemmli solution by incubation at 100°C for 5 min. Samples were then centrifuged at 15,000× *g* for 15 min, and the supernatants were heated at 100°C for 5 min before electrophoresis.

## Results

All the atypical cases detected in France by the surveillance program were initially identified by an ELISA rapid diagnosis test (TeSeE Bio-Rad). The brain stem samples in our atypical scrapie panel (n = 54) invariably gave negative results with modified SAF (Scrapie-associated Fibrils) Immunoblot (*10*,*15*). However, the 51 sheep and 3 goat isolates analyzed gave positive results with the highly sensitive WB method (TeSeE Bio-Rad), which used the classic pK concentration for sample digestion and Sha31 MAb for PrPres detection.

### PrP Genetics of Classic and Atypical Cases

The *prnp* genotypes at codons 136, 141, 154, and 171 are shown in the Table. Most (87.8%) samples from sheep with classic scrapie were observed in genotypes with combinations of the ARQ, ARH, and VRQ alleles. A small proportion (12%) of classic scrapie cases were observed in AF_141_RQ carriers, but no classic scrapie was found in animals carrying the AHQ or ARR allele.

A large proportion (82.3%) of animals in the atypical sheep scrapie group carried the AF_141_RQ (n = 35, 68.6%) or AHQ (n = 7, 13.7%) allele. An unusually high proportion of atypical cases were ARR heterozygous (n = 20, 39.2%), but in 16 cases the ARR allele was associated with either the AF_141_RQ or AHQ allele. Six of the atypical cases were ARR/ARR homozygous (11.8%).

Only 3 of the atypical cases had genotypes combining the ARQ, ARH, and VRQ alleles association, which was strikingly different from the group of classic sheep scrapie cases. Similarly, only 4 animals (7.8%) carried the highly susceptible VRQ allele, and in all cases this was associated with the AF_141_RQ allele. Two of the 3 goats with atypical cases carried the AHQ allele (1 homozygous and 1 heterozygous), whereas none of the 60 sheep with cases of classic scrapie carried the AHQ allele (Table). Taken together, these data strongly suggest that AHQ and AF_141_RQ animals are more susceptibile to atypical scrapie than to classic scrapie.

### PrPres WB Pattern of Atypical Scrapie and Nor98 Isolates

**Figure 1:**
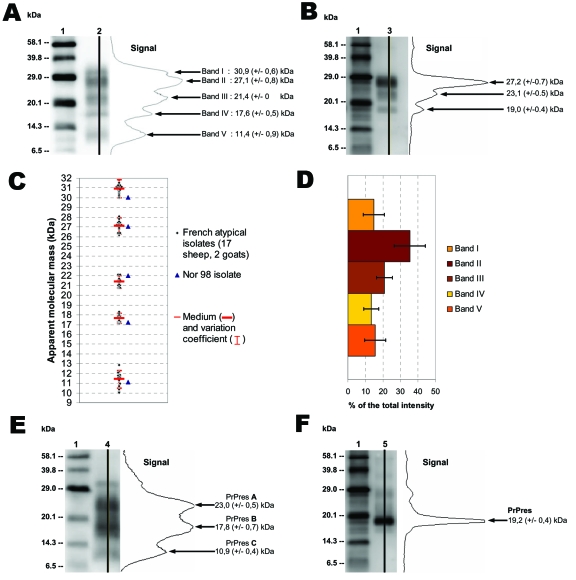
Atypical scrapie and Nor98 isolates PrPres Western blot pattern. Western blot (WB) profile in atypical (A, lane 2) and classic (B, lane 3) scrapie isolates with curves of chemiluminescence measured along the lane and corresponding apparent MWs (MWs), assessed by Bio-Rad Quantity One software analysis after signal capture using Versa Doc5000. Molecular weight (MW) standard (lanes 1). WB profiles of French atypical isolates in sheep (n = 17) and goats (n = 2) were compared with those of a Nor98 isolate. Apparent molecular masses (C) and proportions (D) of bands I to V were assessed from 3 independent runs for each sample by Bio-Rad Quantity One software analysis after signal capture using Versa Doc5000. Apparent MWs are measures for each of the atypical scrapie isolates, and proportions of bands are the means and standard deviations in the 19 atypical scrapie isolates. WB profiles of PrPres after PNGase deglycosylation with curves of chemiluminescence in atypical (E, lane 4) and classic (F, lane 5) scrapie isolates. Apparent MWs were estimated by comparison with a MW standard (lanes 1) from 10 independent runs.

All the atypical isolates showed a complex multiband pattern that differed dramatically from the 3-band pattern observed in classic scrapie (Figure 1A, B). Five major bands (designated I to V according to increasing electromobility) could be distinguished in all atypical cases, irrespective of genotype or species (sheep or goat); in all 54 cases, a V band was clearly observed around 11 kDa.

Twenty isolates, from 17 sheep with various genotypes, 2 goats, and 1 Nor98 sheep (Table), were subjected to repeated electrophoresis to measure the bands’apparent from. According to our measurements, the patterns from the 19 French atypical cases were very similar to each other and indistinguishable from those from the Norwegian Nor98 isolate (Figure 1C).

However, a slight variability could be observed in the apparent MWs between cases. Variations of the WB method were further examined in repeated runs of PrPres isolated from a classic scrapie isolate to assess their possible significance. The analysis of 15 different runs of such a sample showed a variation coefficient (standard deviation divided by mean) of 2.1% (±0.1%) for the 3 bands. The observed variations in the individual measures for atypical scrapie samples were 2.0% (±1.0%) for bands I to IV and 4.5% (±3.0%) for band V. These findings strongly suggest that the observed variations between the different samples were probably due to the method rather than to significant differences between samples.

**Figure 2:**
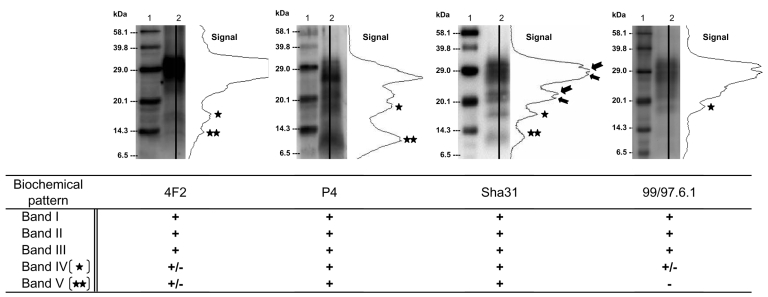
Western blot profiles of PrPres in an atypical scrapie isolate (lane 2) detected by using N-terminal (4F2, P4), central (Sha31), or C-terminal (99/97.6.1) monoclonal antibodies. Molecular weight (MW) standard (lane 1). Immunoreactivities obtained with each antibody on 10 different atypical scrapie isolates are indicated (+, strong, ±, low, –, absent).

The proportion of total PrPres WB signal represented by each band in the same panel of 19 French atypical cases was measured (Figure 1D**)**. Band II was significantly more intense (mean 35%) than band III (mean 20%) or bands I, IV, and V (means 10%–15%). Two individual peaks could be identified in bands II and III in runs and lanes with the highest resolution. These 2 peaks were located at 28.5 (±0.6) and 26.6 (±0.55) kDa in band II and at 22.5 (±0.8) and 20.9 (±0.4) kDa in band III (Figure 2, small arrows).

### PrPres Deglycosylation and Epitope Mapping

Deglycosylations experiments with PNGase before WB analysis were then conducted to investigate the origin of this complex banding pattern. Because of the limited amount of field-collected material (brain stem only) and their low PrPres levels, only 6 atypical sheep isolates and 1 Nor98 isolate could be investigated. A similar pattern of 3 bands at 23.0 (±0.5), 17.8 (±0.7), and 10.9 (±0.4) kDa (referred to as A, B, and C forms, respectively) (Figure 1E) was observed in all 7 samples. PNGase treatment of classic scrapie cases resulted in a single band at 19.2 (±0.4) kDa (Figure 1F), which is consistent with already published data.

The biochemical pattern was characterized by epitope mapping of PrPres using 4F2 (62–93), P4 (93–99), Sha 31 (148–155), and 99/97.6.1 (221–224) (Figure 2). Bands I to III were strongly recognized by all 4 MAbs in our panel, which indicated that the 3 bands at least contained the 85– to 155–amino acid sequence. Band IV was clearly recognized by P4 and Sha31 antibodies and faintly by 4F2 and 99/97.6.1. Band V was not recognized by the more C-terminal 99/97.6.1 antibody, which suggests that this band corresponds to a C-terminal cleaved PrPres fragment but was labeled by the 3 other MAbs, although more weakly by the 4F2 antibody.

## Discussion

In this study, we characterized a series of TSE isolates from French sheep and goats originally classified as having atypical scrapie cases (*10*) on the basis of discrepancies between rapid diagnostic tests and confirmatory methods used to detect PrPres in brain stem samples. Similar discrepancies had been observed in the early description of so-called Nor98 scrapie isolates (*8*). The long-debated hypothesis that atypical cases and Nor98 cases could be only artifacts and not true TSE was recently ruled out by the successful transmission of 10 of these French atypical isolates and 3 Nor98 isolates to transgenic mice overexpressing the ovine PrP (V_136_ R_154_ Q_171_ allele) (*16*). Similarly, PrPres could be detected by using the TeSeE Bio-Rad WB method for all the French atypical and Nor98 cases studied here.

### Original PrPres WB Signature

**Figure 3:**
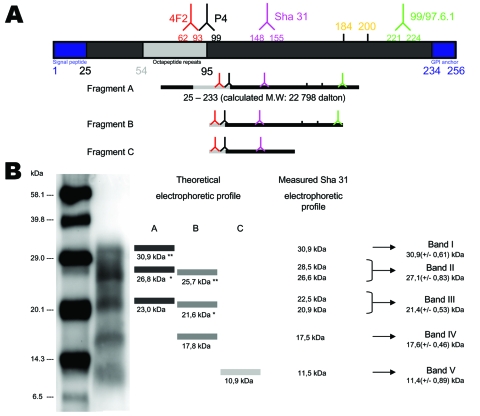
A) Schematic representation of ovine PrPc with location of epitopes recognized by the monoclonal antibodies used during the study and approaching sizes of PrPres fragments in atypical scrapie and Nor98 isolates. Theoretical apparent MWs of PrP fragments were calculated, by using those of each amino acid included in the known ARQ sheep PrP sequence, according to Sambrook and Russell (*17*). B) Interpretation of PrPres Western blot (WB) profiles in atypical scrapie and Nor98 isolates. Theoretical WB profile shows the expected apparent molecular masses of glycosylated PrP forms estimated by addition of 3.8 (*, monoglycosylated) or 7.9 (**, diglycosylated) kDa to the apparent molecular masses of A and B PrPres forms observed after PNGaseF deglycosylation. Values of 3.8 and 7.9 kDa were estimated from comparisons of glycosylated and unglycosylated forms in a classic scrapie isolate. The Sha31 WB profile included the mean apparent MWs assessed from highest resolution WB analysis (n = 32) and showed 2 separate peaks of maximal intensity in pictograms of signal intensities of bands II and III (19 sheep scrapie isolates).

We found that PrPres showed a unique biochemical signature in all cases (54 French atypical and 4 Nor98 isolates) in comparison to classic scrapie, with 1) a multiband pattern with a characteristic band of low MW (11 kDa) and 2) 3 distinct deglycosylated PrPres forms of 23, 18, and 11 kDa (A, B, and C fragments, respectively). Given the theoretical MW of ≈22.8 kDa of the mature ovine PrP protein, the results obtained with all 4 antibodies in our panel, and the C-terminal 99/97.6.1 epitope undetected from only band V, the following hypothetical sequences of the A, B, and C fragments can be inferred (Figure 3A ;[*17*]). The PrPres fragment A (23 kDa) might correspond to a native (uncleaved or marginally cleaved by pK treatment) PrP fragment. The PrPres fragment B could be N terminally cleaved (nearby 4F2 epitope), as in classic scrapie, although cleavage of the C-terminal end cannot be fully excluded. While this scenario is already suggested by the faint labeling with 99/97.6.1 antibody, it would also be consistent with the observation that, despite the presence of the P4 epitope, PrPres B apparently has a lower mass than PrPres in ovine BSE (*18*). The C fragment (11 kDa) could correspond to an N (nearby 4F2 epitope) and C terminally cleaved PrPres protein.

If one assumes that the PrPres glycosylation process could be similar in classic (+3.8 or +7.9 for monoglycosylation and biglycosylation, respectively) and atypical scrapie cases, the theoretical MWs of the unglycosylated, monoglycosylated, and biglycosylated forms that could be derived from A, B, and C fragments can be reconstituted and compared with the banding pattern observed with different antibodies, such as Sha31 MAb (Figure 3B). Glycosylations of the 23-kDa A form would result in bands at 26.8 kDa and 30.9 kDa and those of the 18-kDa B form in bands at 21.6 and 25.7 kDa. These forms are consistent with bands I to IV, as well as with the presence of 2 distinct PrP forms detectable in both band II and band III in WB with the highest resolution (Figure 3B). The absence of detectable bands at 15 kDa and 19 kDa with any of the antibodies tested could suggest that the C form would only be present in the unglycosylated form. This lack of glycosylation would be consistent with a C-terminal cleavage of this PrP form upstream from the N-glycosylation sites (amino acids 184 and 200). Taken together, these hypotheses could explain the unique WB pattern identified in all the French atypical and Nor98 isolates studied here (Figure 3B). However, other hypotheses, such as the existence of random pK-digested fragments resulting in 3 major PrPres with variable sequences after deglycosylation, cannot be fully excluded.

These hypotheses need to be considered in the light of results recently published by Klingeborn et al. (*19*). After purification of PrPres, including a pK treatment at 100 μg/mL for 1 h at 37°C, and concentration by precipitation with trichloroacetic acid, these authors detected 2 PrPres products at 7 kDa (Nor98-PrP7) and 14 kDa (PrP-CTF14) in Nor98 case isolates from Swedish sheep. Importantly, 1 of these Swedish Nor98 cases was investigated in a laboratory taking part in this study (that of S.L. Benestad) and showed the same pattern with the band of low MW at 11 kDa with TeSeE Bio-Rad WB. Differences in the apparent molecular masses between the isolates from the 2 studies could result, at least in part, from the different methods used for PrPres purification and concentration, when one considers the high pK sensitivity of PrPres in atypical scrapie (*10*,*12*). The choice of antibodies could also contribute to the observed differences. For instance, in our study, band V consistently showed an apparently lower molecular mass (9–10 kDa), with P4 antibody (antibody used in the Klingeborn et al. study). Therefore, fragments C (11 kDa) and B (18 kDa) could correspond to Nor98-PrP7 and PrP-CTF14, respectively, in the harsher pK conditions used by Klingeborn et al. PrPres fragment A (23 kDa) was not observed by Klingeborn et al., which suggests that this PrPres form might be completely digested or transformed into fragments of Nor98-PrP7, PrP-CTF14, or both. Despite minor differences, the results of the 2 studies in regard to the particular molecular features of atypical scrapie/Nor98 isolates are consistent.

The presence of a PrPres fragment with an apparently low molecular mass, which is a salient feature of atypical and Nor98 cases, has already been reported in several human diseases. N terminally truncated PrPres migrating to either 12 kDa or 13 kDa have been reported in some sporadic Creutzfeldt-Jakob disease cases (*20*). The identification of a low-MW fragment cleaved at both C- and N-terminal ends of the prion protein has even been described as the hallmark of Gerstmann-Sträussler-Scheinker syndrome in humans (*2*,*21*–*24*). However, even if these atypical isolates appear to have similarities with those from rare human prion diseases, atypical cases of scrapie are not rare in sheep and goats and, in some countries, are more frequent than the classic disease.

### Biodiversity in Atypical Cases

The isolates from the 54 atypical cases we investigated here, which included a large panel of different PrP genotypes and 2 species (sheep and goat), possessed a unique biochemical signature, indistinguishable from that of the Nor98 isolates. All of the isolates from the French atypical and Nor98 cases that were transmitted to Tg338 ovine transgenic mice also shared the same biologic signature, with comparable incubation periods, clinical signs, lesion profiles, and PrPres deposits patterns in the central nervous system (*16*). Moreover, the PrPres biochemical signature in the inoculated Tg338 was strikingly comparable to that observed in the present study. Taken together, these data strongly suggest that the prions involved in atypical (sheep and goat) and Nor98 cases are in fact a unique TSE agent. However, because our cases were obtained from only 2 countries, whereas atypical cases have been identified throughout Europe (*12*,*25*–*29*), further studies are required before definitive conclusions can be drawn. Nevertheless, the lack of diversity in our panel, combined with the identity with Nor98 cases, suggests that the biodiversity of the TSE agents of atypical scrapie is not large.

### Allelic Tropism in Atypical Cases

Although polymorphisms associated with classic scrapie have been widely documented (*4*,*6*), both the atypical and Nor98 cases (*9*) seemed to deviate from the established concepts of classic scrapie. In our panel, an obviously high susceptibility seemed to be associated with the AHQ and AF_141_RQ alleles, whereas the VRQ allele was poorly represented. Similar observations were reported for Nor98 cases (*8*,*9*).

However, a proper analysis of the risk associated with each genotype in atypical cases will require much more data than those presented here. Indeed, all our atypical case data were collected through active surveillance network, by using a particular rapid diagnosis test. These affected animals would need to be matched for breed, age, and population (detection within the same active surveillance program with similar tests) to permit an appropriate risk factor analysis and comparison of susceptibility with animals with classic scrapie. Moreover, the distribution of F_141_ within each breed is currently unknown. This work is ongoing in France, and results should be presented soon.

The involvement of ARR/ARR genotype animals not only in our study (6 cases) but also in several EU countries (*11*,*30*) is also of some concern. Based on the observed resistance to TSE in homozygous ARR animals, a breeding program for resistance to scrapie and BSE has been implemented in several EU countries to control human exposure to TSE risk. This unusual susceptibility of small ruminants believed to be genetically resistant to TSE could lead to a reevaluation of such a policy. In this context, determining the distribution of infectivity in different tissues of affected animals and whether or not atypical scrapie is naturally transmissible between animals within affected flocks would also be helpful.

## Conclusion

Our data provide new information about the recently described atypical cases of TSE. These cases appear to be associated with a novel PrPres biochemical pattern; they shared similarities with some rare prion diseases in humans and were clearly distinct from classic scrapie or BSE. This potential similarity in PrPres formation mechanisms with some other rare prion diseases in humans is intriguing. However, the unusual properties of these atypical cases illustrate our decades of underestimating the biodiversity of TSEs in small ruminants and the consequences. This finding should lead to a general reexamination of our conceptual approach in the control of TSEs in small ruminants.

## References

[1] McKinley MP, Bolton DC, Prusiner SB. A protease-resistant protein is a structural component of the scrapie prion. Cell. 1983;35:57–62. 10.1016/0092-8674(83)90207-6 6414721

[2] Tagliavini F, Lievens PM, Tranchant C, Warter JM, Mohr M, Giaccone G, A 7-kDa prion protein (PrP) fragment, an integral component of the PrP region required for infectivity, is the major amyloid protein in Gerstmann-Straussler-Scheinker disease A117V. J Biol Chem. 2001;276:6009–15. 10.1074/jbc.M007062200 11087738

[3] Clouscard C, Beaudry P, Elsen JM, Milan D, Dussaucy M, Bounneau C, Different allelic effects of the codons 136 and 171 of the prion protein gene in sheep with natural scrapie. J Gen Virol. 1995;76:2097–101. 10.1099/0022-1317-76-8-2097 7636494

[4] Hunter N, Foster JD, Goldmann W, Stear MJ, Hope J, Bostock C. Natural scrapie in a closed flock of Cheviot sheep occurs only in specific PrP genotypes. Arch Virol. 1996;141:809–24. 10.1007/BF01718157 8678828

[5] Baylis M, Chihota C, Stevenson E, Goldmann W, Smith A, Sivam K, Risk of scrapie in British sheep of different prion protein genotype. J Gen Virol. 2004;85:2735–40. 10.1099/vir.0.79876-0 15302967

[6] Hunter N, Goldmann W, Foster JD, Cairns D, Smith G. Natural scrapie and PrP genotype: case-control studies in British sheep. Vet Rec. 1997;141:137–40. 9280041 10.1136/vr.141.6.137

[7] Andreoletti O, Morel N, Lacroux C, Rouillon V, Barc C, Tabouret G, Bovine spongiform encephalopathy agent in spleen from an ARR/ARR orally exposed sheep. J Gen Virol. 2006;87:1043–6. 10.1099/vir.0.81318-0 16528056

[8] Benestad SL, Sarradin P, Thu B, Schonheit J, Tranulis MA, Bratberg B. Cases of scrapie with unusual features in Norway and designation of a new type, Nor98. Vet Rec. 2003;153:202–8. 12956297 10.1136/vr.153.7.202

[9] Moum T, Olsaker I, Hopp P, Moldal T, Valheim M, Benestad SL. Polymorphisms at codons 141 and 154 in the ovine prion protein gene are associated with scrapie Nor98 cases. J Gen Virol. 2005;86:231–5. 10.1099/vir.0.80437-0 15604451

[10] Buschmann A, Biacabe AG, Ziegler U, Bencsik A, Madec JY, Erhardt G, Atypical scrapie cases in Germany and France are identified by discrepant reaction patterns in BSE rapid tests. J Virol Methods. 2004;117:27–36. 10.1016/j.jviromet.2003.11.017 15019257

[11] Buschmann A, Luhken G, Schultz J, Erhardt G, Groschup MH. Neuronal accumulation of abnormal prion protein in sheep carrying a scrapie-resistant genotype (PrPARR/ARR). J Gen Virol. 2004;85:2727–33. 10.1099/vir.0.79997-0 15302966

[12] Everest SJ, Thorne L, Barnicle DA, Edwards JC, Elliott H, Jackman R, Atypical prion protein in sheep brain collected during the British scrapie-surveillance programme. J Gen Virol. 2006;87:471–7. 10.1099/vir.0.81539-0 16432036

[13] Feraudet C, Morel N, Simon S, Volland H, Frobert Y, Creminon C, Screening of 145 anti-PrP monoclonal antibodies for their capacity to inhibit PrPSc replication in infected cells. J Biol Chem. 2005;280:11247–58. 10.1074/jbc.M407006200 15618225

[14] Thuring CM, van Keulen LJ, Langeveld JP, Vromans ME, van Zijderveld FG, Sweeney T. Immunohistochemical distinction between preclinical bovine spongiform encephalopathy and scrapie infection in sheep. J Comp Pathol. 2005;132:59–69. 10.1016/j.jcpa.2004.06.004 15629480

[15] Madec JY, Belli P, Calavas D, Baron T. Efficiency of Western blotting for the specific immunodetection of proteinase K–resistant prion protein in BSE diagnosis in France. Vet Rec. 2000;146:74–6. 10674695 10.1136/vr.146.3.74

[16] Le Dur A, Beringue V, Andreoletti O, Reine F, Lai TL, Baron T, A newly identified type of scrapie agent can naturally infect sheep with resistant PrP genotypes. Proc Natl Acad Sci U S A. 2005;102:16031–6. 10.1073/pnas.0502296102 16239348 PMC1276041

[17] Sambrook J, Russell DW. Codons and amino acids. Molecular cloning-laboratory manuals. 3rd edition. New York: Cold Spring Harbor; 2001. p. A7–9.

[18] Stack MJ, Chaplin MJ, Clark J. Differentiation of prion protein glycoforms from naturally occurring sheep scrapie, sheep-passaged scrapie strains (CH1641 and SSBP1), bovine spongiform encephalopathy (BSE) cases and Romney and Cheviot breed sheep experimentally inoculated with BSE using two monoclonal antibodies. Acta Neuropathol. 2002;104:279–86. 12172914 10.1007/s00401-002-0556-2

[19] Klingeborn M, Wik L, Simonsson M, Renstrom LH, Ottinger T, Linne T. Characterization of proteinase K-resistant N- and C-terminally truncated PrP in Nor98 atypical scrapie. J Gen Virol. 2006;87:1751–60. 10.1099/vir.0.81618-0 16690942

[20] Zou WQ, Capellari S, Parchi P, Sy MS, Gambetti P, Chen SG. Identification of novel proteinase K-resistant C-terminal fragments of PrP in Creutzfeldt-Jakob disease. J Biol Chem. 2003;278:40429–36. 10.1074/jbc.M308550200 12917418

[21] Tagliavini F, Prelli F, Ghiso J, Bugiani O, Serban D, Prusiner SB, Amyloid protein of Gerstmann-Straussler-Scheinker disease (Indiana kindred) is an 11 kd fragment of prion protein with an N-terminal glycine at codon 58. EMBO J. 1991;10:513–9. 1672107 10.1002/j.1460-2075.1991.tb07977.x PMC452678

[22] Parchi P, Chen SG, Brown P, Zou W, Capellari S, Budka H, Different patterns of truncated prion protein fragments correlate with distinct phenotypes in P102L Gerstmann-Straussler-Scheinker disease. Proc Natl Acad Sci U S A. 1998;95:8322–7. 10.1073/pnas.95.14.8322 9653185 PMC20974

[23] Ghetti B, Piccardo P, Spillantini MG, Ichimiya Y, Porro M, Perini F, Vascular variant of prion protein cerebral amyloidosis with tau-positive neurofibrillary tangles: the phenotype of the stop codon 145 mutation in PRNP. Proc Natl Acad Sci U S A. 1996;93:744–8. 10.1073/pnas.93.2.744 8570627 PMC40125

[24] Ghetti B, Piccardo P, Frangione B, Bugiani O, Giaccone G, Young K, Prion protein amyloidosis. Brain Pathol. 1996;6:127–45. 10.1111/j.1750-3639.1996.tb00796.x 8737929

[25] Orge L, Galo A, Machado C, Lima C, Ochoa C, Silva J, Identification of putative atypical scrapie in sheep in Portugal. J Gen Virol. 2004;85:3487–91. 10.1099/vir.0.80246-0 15483267

[26] Gavier-Widen D, Noremark M, Benestad S, Simmons M, Renstrom L, Bratberg B, Recognition of the Nor98 variant of scrapie in the Swedish sheep population. J Vet Diagn Invest. 2004;16:562–7. 15586572 10.1177/104063870401600611

[27] Epstein V, Pointing S, Halfacre S. Atypical scrapie in the Falkland Islands. Vet Rec. 2005;157:667–8. 16299372 10.1136/vr.157.21.667-c

[28] De Bosschere H, Roels S, Benestad SL, Vanopdenbosch E. Scrapie case similar to Nor98 diagnosed in Belgium via active surveillance. Vet Rec. 2004;155:707–8. 15605538 10.1136/vr.155.22.707

[29] Onnasch H, Gunn HM, Bradshaw BJ, Benestad SL, Bassett HF. Two Irish cases of scrapie resembling Nor98. Vet Rec. 2004;155:636–7. 15573787 10.1136/vr.155.20.636

[30] De Bosschere H, Roels S, Dechamps P, Vanopdenbosch E. TSE detected in a Belgian ARR-homozygous sheep via active surveillance. Vet J. 2005; [Epub ahead of print]. 10.1016/j.tvjl.2005.07.014 16169265

